# Expression Atlas of FGF and FGFR Genes in Pancancer Uncovered Predictive Biomarkers for Clinical Trials of Selective FGFR Inhibitors

**DOI:** 10.1155/2020/5658904

**Published:** 2020-06-03

**Authors:** Yuan Li, Long Wu, Weiping Tao, Dawei Wu, Fei Ma, Ning Li

**Affiliations:** ^1^Department of Oncology, Renmin Hospital of Wuhan University, Wuhan, China; ^2^Department of Thoracic Surgery, National Cancer Center/National Clinical Research Center for Cancer/Cancer Hospital, Chinese Academy of Medical Sciences and Peking Union Medical College, Beijing, China; ^3^Department of Good Clinical Practice Center, National Cancer Center/National Clinical Research Center for Cancer/Cancer Hospital, Chinese Academy of Medical Sciences and Peking Union Medical College, Beijing, China; ^4^Department of Medical Oncology, National Cancer Center/National Clinical Research Center for Cancer/Cancer Hospital, Chinese Academy of Medical Sciences and Peking Union Medical College, Beijing, China

## Abstract

**Background:**

Clinical trials based on FGFR mutation or amplification as a druggable target of FGFR inhibitors have produced disappointing clinical outcomes. Therefore, the identification of predictive biomarkers for FGFR-targeted agents has remained a crucial issue.

**Methods:**

Expression profiles of FGFs and FGFRs in 8,111 patients with 24 types of solid tumors and 879 tumor cell lines along with drug sensitivity data were obtained and followed by integrative bioinformatics analysis.

**Results:**

FGFs and FGFRs were frequently dysregulated in pancancer. Most of the expression of FGFs and FGFRs were significantly associated with overall survival in at least two cancer types. Moreover, tumor cell lines with high FGFR1/3 expression were more sensitive to FGFR inhibitor PD173074, especially in breast, liver, lung and ovarian cancer. The predicted positive ratios of FGFR1-4 were generally over 10% in most tumor types, especially in squamous cell carcinoma. High positive FGFR1 or 3 expression ratios were predicted in cholangiocarcinoma (58%), followed by bladder cancer (42%), endometrial carcinoma (35%), and ovarian cancer (34%).

**Conclusions:**

FGFR expression was a promising predictive biomarker for FGFR inhibition response in clinical trials, and different combinations of FGFR genes should be used in screening for patients in certain tumor types.

## 1. Introduction

Fibroblast growth factors (FGFs) and their transmembrane tyrosine kinase receptors (FGFRs) play vital roles in important biological processes in homeostasis [1]. In human, the FGFs contain 22 members, and canonical FGFs can bind and activate FGFRs, triggering an intracellular signaling cascade that mediates their biological activities [2]. FGFRs are encoded by four distinct genes, termed FGFR1-4, that display overlapping affinities/specificities for the various FGFs [3]. In cancer, FGFR signaling represents key players in the complex crosstalk within tumor microenvironment by autocrine and paracrine functions, resulting in angiogenesis, inflammation, tumor growth, and drug resistance [4–6]. Given the strong link between aberrant FGFR signaling and carcinogenesis, inhibiting FGFRs, rather than diverse FGFs, may exert a profound influence on the growth of FGF/FGFR-driven tumors. Therefore, FGFR inhibition appears to be an innovative approach for new cancer therapies.

To date, several selective and nonselective FGFR tyrosine kinase inhibitors (TKIs) have been developed and several specific orally bioavailable small-molecule inhibitors of FGFR are currently in clinical development [7]. For example, dovitinib is an oral TKI targeting FGFR1-3 [8]. However, a phase II study (NCT01861197) of dovitinib in lung squamous cell carcinoma (LUSC) patients with FGFR1 amplification resulted in only a limited clinical activity [9]. Other FGFR-targeted TKIs such as AZD4547 and BGJ398 have produced disappointing clinical outcomes in FGFR-amplified malignancies, raising an important issue whether traditional genomic variants such as FGFR amplification are powerful biomarkers to FGFR-targeted TKIs [10, 11]. Therefore, the identification of predictive biomarkers for FGFR-targeted TKIs has great potential in clinical trials.

Unlike genomic variants in FGFR which had been summarized by a number of reviews, the clinical relevance of FGF and FGFR expression had been ignored with few systematic analyses across different solid tumor types. Here, we reported the expression atlas of FGF and FGFR in pancancer from the perspective of potential application in clinical trials.

## 2. Methods and Materials

### 2.1. Data Curation

Genomic variants of FGFR in pancancer were analyzed and plotted by the cBioPortal for Cancer Genomics (http://www.cbioportal.org/). RNA-Seq data of a total of 8,111 patients with 24 types of solid tumor were downloaded from The Cancer Genome Atlas (TCGA) data portal (https://portal.gdc.cancer.gov/). Expression of FGFR and drug sensitivity data (IC50 values) of PD173074 in 879 tumor cell lines were downloaded from the Genomics of Drug Sensitivity in Cancer Project (GDSC, https://www.cancerrxgene.org/) [12].

### 2.2. Differential Expression Analysis and Positive Ratio Prediction

Differential expression analysis between tumor and normal tissues was tested by the Wilcoxon test. Some tumor types, including ACC, OV, and LGG, were excluded since there were no normal tissues in these tumor types. The detailed sample sizes for each included tumor types are listed in Table 1.

For positive expression prediction, the calculation was based on data from a phase I expansion clinical trial of Rogaratinib (BAY1163877), in which over 40% were found to be FGFR1 or 3 positive in a total of 219 bladder cancer patients using an RNA in situ hybridization (RNA-ISH) test (RNASCOPE) [13]. Therefore, we determined the cutoff value of 4,220 by setting the FGFR1- or 3-positive ratios in bladder urothelial carcinoma (BLCA) of 42% and calculated the positive ratios in other tumor types with the same cutoff value.

### 2.3. Drug Sensitivity Prediction

The GDSC database comprises drug sensitivity data for over 200 anticancer drugs across over 1,000 cancer cell lines [12]. Among them, PD173074, targeting FGFR1 and 3, were tested in a total of 879 tumor cell lines. We tested if FGFR1 or 3 expression was a biomarker for PD173074 sensitivity by correlation analysis of IC50 values and FGFR1 or 3 expression data among the matched tumor cell lines.

### 2.4. Survival Analysis

Clinical parameters of the TCGA cohort were also downloaded from the TCGA database. Only patients with fully characterized tumors and with at least 30 days of overall survival were included in the survival analysis. For each parameter, the patients were divided into two groups with the cutoff value determined by survminer package (version 0.4.2) in R. Then, the association between gene expression and overall survival was carried out using univariate Cox regression.

### 2.5. Statistical Analysis

Differential expression analysis was tested by the Wilcoxon test. The survival curves were compared using the Kaplan-Meier method and the log-rank test. Correlation analysis was tested by the Pearson method. In GDSC, the ANOVA calculates a *P* value to determine the significance of each drug interaction. A *P* value threshold of <10^−3^ and a false discovery rate (Benjamini-Hochberg method) threshold equal to 25% were used to call significant associations across all the performed analyses. All tests were two-sided, and a *P* value of less than 0.05 was considered statistically significant unless stated otherwise. Data were analyzed using R (version 3.4.4).

## 3. Results

### 3.1. FGF and FGFR Genes Were Frequently Dysregulated in Pancancer

**Figure 1:**
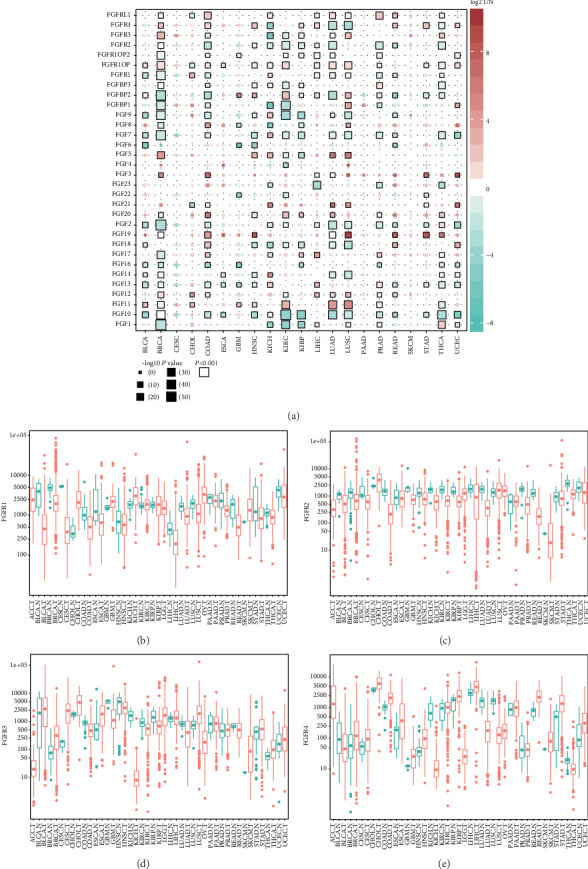
FGF and FGFR genes were frequently dysregulated in pancancer. (a) The color of the boxes indicated the log2 fold change of gene expression in tumor (T) compared with normal (N) tissues, the size represented significance, and the black border means *P* < 0.001. (b–e) Column plots of FGFR1-4 expression in pancancer; red represented tumor while green represented normal tissues.

We compared the expression of FGF family genes between tumor tissues and normal tissues (if available), and the results are summarized in Figure 1(a). Most of the FGF family genes, except genes that were rarely expressed, were significantly dysregulated in at least three tumor types (Figure 1(a), *P* < 0.001). Almost all tumor types, especially in breast invasive carcinoma (BRCA), colon adenocarcinoma (COAD), head and neck squamous cell carcinoma (HNSC), lung adenocarcinoma (LUAD), and LUSC, showed aberrant expression of FGF family genes except for cervical and endocervical cancers (CESC), pancreatic adenocarcinoma (PAAD), and skin cutaneous melanoma (SKCM) (Figure 1(a)). It is worth noting that there were few normal samples in some tumor types, such as SKCM. Similar patterns are shown in Supplementary Figure S1 by differential expression analysis with matched tumor and normal samples.

In detail, FGF1 and FGF10 were downregulated in most of the tumor types whereas FGF3, FGF5, FGF11, FGF19, FGF20, and FGF21 were upregulated in most of the tumor types. Other FGF family genes were downregulated in some of the tumor types whereas upregulated in other tumor types (Figure 1(a)). Moreover, FGFR1-4 were also widely dysregulated among many tumors. FGFR1 was significantly downregulated in BLCA and BRCA, whereas it was significantly upregulated in CHOL and KICH (Figures 1(a) and 1(b)). FGFR2 was generally downregulated in most of the tumors, including COAD, kidney renal clear cell carcinoma (KIRC), LUAD, prostate adenocarcinoma (PRAD), and thyroid carcinoma (THCA) (Figures 1(a) and 1(c)). FGFR3 was significantly upregulated in BRCA, LUSC, and THCA and was downregulated in kidney chromophobe (KICH) (Figures 1(a) and 1(d)). FGFR4 was significantly upregulated in BRCA, COAD, HNSC, rectum adenocarcinoma (READ), and stomach adenocarcinoma (STAD) and was downregulated in KICH, LUAD, and LUSC (Figures 1(a) and 1(e)).

### 3.2. FGF and FGFR Genes Showed Mixed Prognostic Value in Pancancer

**Figure 2:**
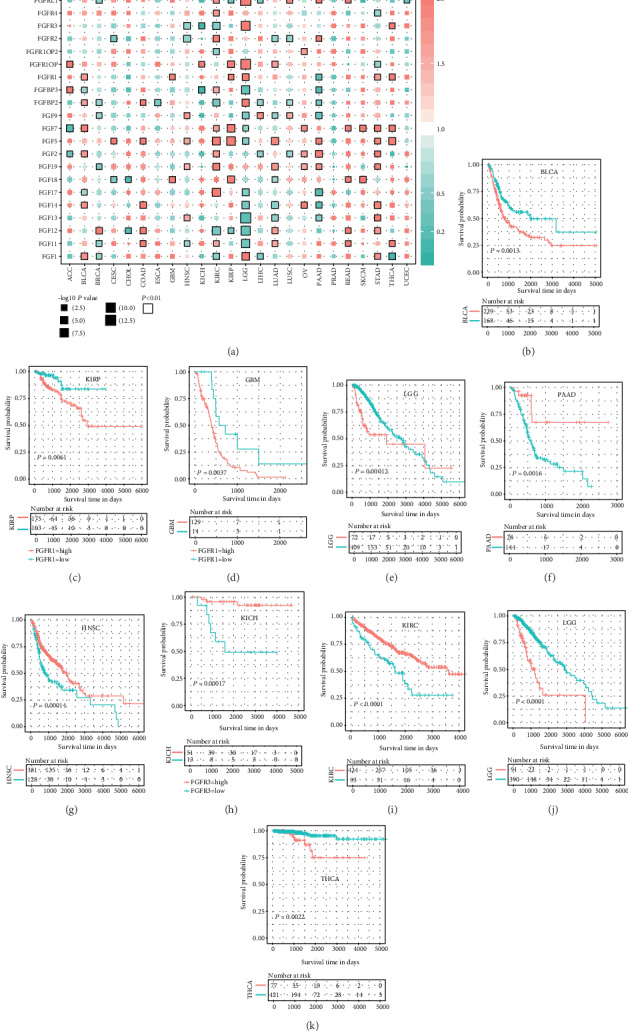
FGF and FGFR genes showed mixed prognostic value in pancancer. (a) Survival analysis results in pancancer showing hazard ratios (HR) and significance. (b–k) Kaplan-Meier plots of FGFR1 or 3 in specific tumor types.

In survival analysis, FGF and FGFR genes showed a mixed prognostic value in pancancer (Figure 2(a)). Almost all the FGF and FGFR genes were significantly associated with at least two cancer types, except that a few genes were rarely expressed and undetectable in most of the tumor samples (not shown). High expression of one gene might indicate poor overall survival in some tumor types and good overall survival in other tumor types (Figure 2(a)). Taking FGFR1-4 as an example, FGFR1 was an adverse prognostic factor in BLCA, kidney renal papillary cell carcinoma (KIRP), glioblastoma multiforme (GBM), and LGG, whereas it was a favorable prognostic factor in PAAD (Figures 2(b)–2(f)). Similarly, HNSC, KICH, and KIRC patients with high FGFR3 expression and LGG and THCA patients with low FGFR3 expression had better overall survival (Figures 2(g)–2(k)). In Supplementary Figure S2, FGFR2 and FGFR4 were both adverse prognostic factors in KIRC, whereas FGFR2 were favorable prognostic factors in CESC, HNSC, LUSC, and LUAD.

### 3.3. Cell Lines with High FGFR1/3 Expression Were More Sensitive to PD173074

**Figure 3:**
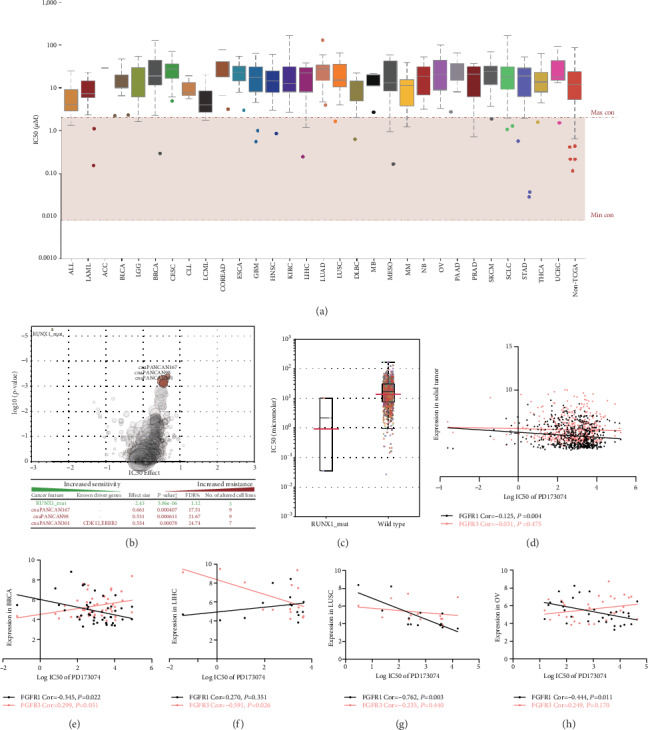
Cell lines with high FGFR1/3 expression were more sensitive to PD173074. (a) IC50 values of PD173074 in pancancer. Each box contains 2nd and 3rd quartile data. Whiskers go up to 1.5 times interquartile range (log-transformed). (b, c) Cell lines with RUNX1 mutation were more sensitive to PD173074. (d–h) FGFR1 or 3 expression was significantly correlated with IC50 values of PD173074.

**Figure 4:**
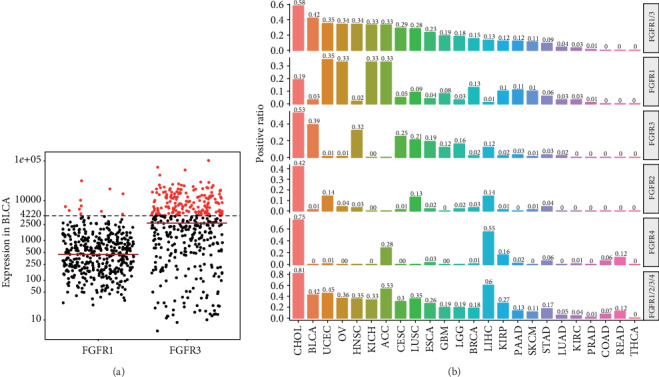
High positive ratios of FGFR1-4 were predicted in certain types of cancer. (a) The cutoff value was set to 4,220 to reach FGFR1 or 3 positive ratio of 42%, which was curated from preliminary results from an ongoing clinical trial (NCT01976741) in advanced bladder cancer patients prescreened for FGFR1 or 3 mRNA expression levels by RNA-ISH. Here, we assume that FGFR1- or 3-positive ratio by RNA-ISH is positively correlated with RSEM value by RNA-Seq in TCGA. (b) Predicted positive ratios of FGFR1-4 in pancancer.

PD173074 is a potent FGFR small-molecule kinase inhibitor targeting FGFR1 and 3 [14]. Among the solid tumor types, squamous cell carcinoma seemed to be more sensitive than adenocarcinoma; HNSC and LUSC cell lines had lower IC50 values than esophageal carcinoma (ESCA) and LUAD, respectively (Figure 3(a)). GDSC analysis showed that RUNX1 mutation would increase cell line sensitivity to PD173074 (Figures 3(b) and 3(c)), whereas CDK12 or ERBB2 mutation would increase cell line resistance to PD173074 (Figure 4(b)). Here, the correlation analysis highlighted that FGFR1 and 3 expression was significantly correlated with PD173074 sensitivity (Figures 3(d)–3(g)). Overall, FGFR1 expression was significantly correlated with IC50 of PD173074 in all solid tumor cell lines (Figure 3(d)). Separately, a significant correlation between FGFR1 expression and IC50 values was also observed in BRCA, LUSC, and OV (Figures 3(e), 3(g), and 3(h)) while FGFR3 was significantly correlated with IC50 values in LIHC (Figure 3(f)). Taken together, besides generic mutations, cell lines with high FGFR1/3 expression were more sensitive to PD173074 in solid tumors, including BRCA, LIHC, LUSC, and OV.

### 3.4. High Positive Ratios of FGFR1-4 Were Predicted in Certain Types of Cancer

Rogaratinib (BAY1163877) is a selective oral inhibitor of FGFR1-4; promising results have been reported from a phase I expansion cohort in advanced bladder cancer patients prescreened for FGFR1 or 3 mRNA expression levels by RNA-ISH (NCT01976741) [13]. In a total of 219 urothelial cancer patients, over 40% of the patients were found to be positive [13]. Here, we assume that FGFR1 or 3 positive ratio by RNA-ISH is positively correlated with RSEM value by RNA-Seq in TCGA. Therefore, we determined the cutoff value of 4,220 by setting the FGFR1 or 3 positive ratio in BLCA of 42% (Figure 4(a)). Moreover, we estimated the positive ratios of FGFR1-4 in all the solid tumors in TCGA. In Figure 4(b), the highest positive ratio of FGFR1 or 3 was in CHOL (58%), followed by BLCA (42%), UCEC (35%), OV (34%), and HNSC (34%). Positive ratios of FGFR1-4 were also presented separately, and the expression pattern of FGFR1-4 varied a lot among the cancer types. Interestingly, the positive ratios of FGFR1-4 in CHOL were all very high while only one or two FGFR genes were positively expressed in other types of tumor. For example, over 30% patients had positive FGFR1 expression in UCEC, OV, KICH, and ACC, whereas the positive ratios of FGFR3 were very low in these tumor types (Figure 4(b)). This phenomenon was also seen in BLCA and HNSC, in which positive expression mainly came from FGFR3 rather than FGFR1 (Figure 4(b)). Compared with FGFR1 and 3, positive ratios of FGFR2 or 4 were generally lower in most of the tumor types, except in CHOL and LIHC (Figure 4(b)). The FGFR expression in individual tumor tissue is presented in Supplementary Figure S3. Taken together, the positive ratios of FGFR1/2/3/4 were generally over 10% in most tumor types, indicating that right combination of FGFR1-4 genes might benefit patient enrollment in clinical trials in certain tumor types (Figure 4(b)). These results provided a general and specific estimation of FGFR1-4-positive ratios and important guidance in choosing the right inclusion criteria and patients in future clinical trials.

## 4. Discussion

Great attention had been paid to genomic alterations of FGF-FGFR in the past decades [15, 16]; we also summarized the genomic mutations and alteration frequency in pancancer from the cBioPortal for Cancer Genomics in Supplementary Figure S4. FGF and FGFR expression remains largely unstudied in clinical application.

At present, some of the nonselective FGFR TKIs, including brivatinib, lenvatinib, regorafenib, ponatinib, and dovitinib [15], have achieved approval for use against several cancer types; however, many of these multi-TKIs are less capable of achieving an efficient FGFR inhibition and also increase side effects. Nowadays, pharmaceutical companies are developing more potent FGFR TKIs. Selective FGFR TKIs include AZD4547, BGJ398, LY2874455, TAS-120, ARQ087, PD173074, JNJ-42756493, BLU9931, DEBIO1347, FGF401, and BAY-1163877 [15]. However, genomic variants including FGFR amplification failed to be powerful biomarkers for FGFR TKI responses in clinical trials [10, 11].

Here, we systematically analyzed the clinical relevance of FGF and FGFR gene expression in pancancer. The results showed that FGF and FGFR genes were frequently dysregulated and significantly associated with overall survival in solid tumors. More importantly, tumor cell lines with high FGFR1/3 expression were more sensitive to FGFR inhibitor PD173074, especially in BRCA, LIHC, LUSC, and OV. RUNX1, CDK12, or ERBB2 mutation might be associated with cell line sensitivity to PD173074. Ultimately, the positive ratios of FGFR1/2/3/4 were generally over 10% in most tumor types. High positive FGFR1 or 3 expression ratios were predicted in CHOL (58%), followed by BLCA (42%), UCEC (35%), OV (34%), and HNSC (34%). The expression pattern of FGFR1-4 varied a lot among all the cancer types. These results provided a general and specific estimation of FGFR1-4-positive ratios and important guidance in choosing the right inclusion criteria and patients in future clinical trials.

Our report highlighted that FGFR gene expression signatures were potential predictors for the response to FGFR TKIs in certain cancer types. A previous study reported that FGFR1 mRNA and protein expression, not gene copy number, predicted FGFR TKI sensitivity across all lung cancer histology [11]. In fact, several ongoing clinical trials of selective FGFR TKIs are using FGFR expression rather than FGFR amplification as screening biomarkers in selecting patients with potential benefits [15]. The selection of patients for treatment based on FGFR mRNA expression levels was feasible and identified drug-sensitive patients with and without underlying DNA alterations [13].

It is worth noting that mixed results were found in the differential expression and survival analysis and not all FGF or FGFR genes were significantly upregulated in tumor tissues and adverse prognosis factors in all solid tumor types. The overall survival may be affected by many factors, including the driven signaling pathways and different therapies recommended currently. These results reminded us that the precise roles of FGF and FGFR genes were cancer-specific and application of FGFR TKIs in cancer should be handled with caution. Besides, our results were based on publicly available data and needed further experimental or clinical validations in the future. More importantly, choosing the right single or combination of FGFR genes as screening biomarkers had a great influence on clinical trial practice. For example, the positive FGFR3 ratios might be high in BLCA whereas positive FGFR4 ratios were high in CHOL and LIHC.

In summary, we provided the expression atlas of FGF and FGFR genes in pancancer and FGFR expression was a promising predictive biomarker for FGFR inhibition response in clinical trials.

## Figures and Tables

**Table 1:** Abbreviations of tumor types and number of RNA sequencing data from TCGA used in this study.

Cancer types	Abbreviation	Tumor	Normal
Adrenocortical carcinoma	ACC	79	NA
Bladder urothelial carcinoma	BLCA	407	19
Breast invasive carcinoma	BRCA	1092	112
Cervical and endocervical cancers	CESC	304	3
Cholangiocarcinoma	CHOL	36	9
Colon adenocarcinoma	COAD	284	41
Esophageal carcinoma	ESCA	183	11
Glioblastoma multiforme	GBM	152	5
Head and neck squamous cell carcinoma	HNSC	519	44
Kidney chromophobe	KICH	66	25
Kidney renal clear cell carcinoma	KIRC	532	72
Kidney renal papillary cell carcinoma	KIRP	290	32
Brain lower grade glioma	LGG	516	NA
Liver hepatocellular carcinoma	LIHC	370	50
Lung adenocarcinoma	LUAD	515	59
Lung squamous cell carcinoma	LUSC	501	51
Ovarian serous cystadenocarcinoma	OV	302	NA
Pancreatic adenocarcinoma	PAAD	178	4
Prostate adenocarcinoma	PRAD	497	52
Rectum adenocarcinoma	READ	94	10
Skin cutaneous melanoma	SKCM	103	1
Stomach adenocarcinoma	STAD	414	35
Thyroid carcinoma	THCA	501	59
Uterine corpus endometrial carcinoma	UCEC	176	24

## Data Availability

The figure and table data used to support the findings of this study are included within the article.

## Supplementary Materials

Supplementary Materials Supplementary Figure S1: differential expression analysis of FGF and FGFR genes with matched tumor and normal samples in pancancer. Supplementary Figure S2: Kaplan-Meier plots of FGFR2 or 4 in specific tumor types. Supplementary Figure S3: the specific expression of FGFR1-4 for each patient in pancancer. Supplementary Figure S4: the mutation frequencies of FGFR1-4 in pancancer from cBioPortal.
