# Supplementary material for: Expression Atlas of FGF and FGFR Genes in Pancancer Uncovered Predictive Biomarkers for Clinical Trials of Selective FGFR Inhibitors
Source: Biomed Res Int. 2020 Jun 3;2020:5658904. doi: 10.1155/2020/5658904 (PMC7293733; doi:10.1155/2020/5658904)
Supplement: Supplementary Materials — Supplementary Figure S1: differential expression analysis of FGF and FGFR genes with matched tumor and normal samples in pancancer. Supplementary Figure S2: Kaplan-Meier plots of FGFR2 or 4 in specific tumor types. Supplementary Figure S3: the specific expression of FGFR1-4 for each patient in pancancer. Supplementary Figure S4: the mutation frequencies of FGFR1-4 in pancancer from cBioPortal. [file 5658904.f1.docx]

**Expression atlas of FGF and FGFR genes in pan-cancer uncovered predictive biomarkers for FGFR inhibition response in clinical trials**

Yuan Li^1,4^, Long Wu^1^, Weiping Tao^1^, Dawei Wu^2^, Fei Ma^3^, Ning Li^4*^

**Supplemental material**


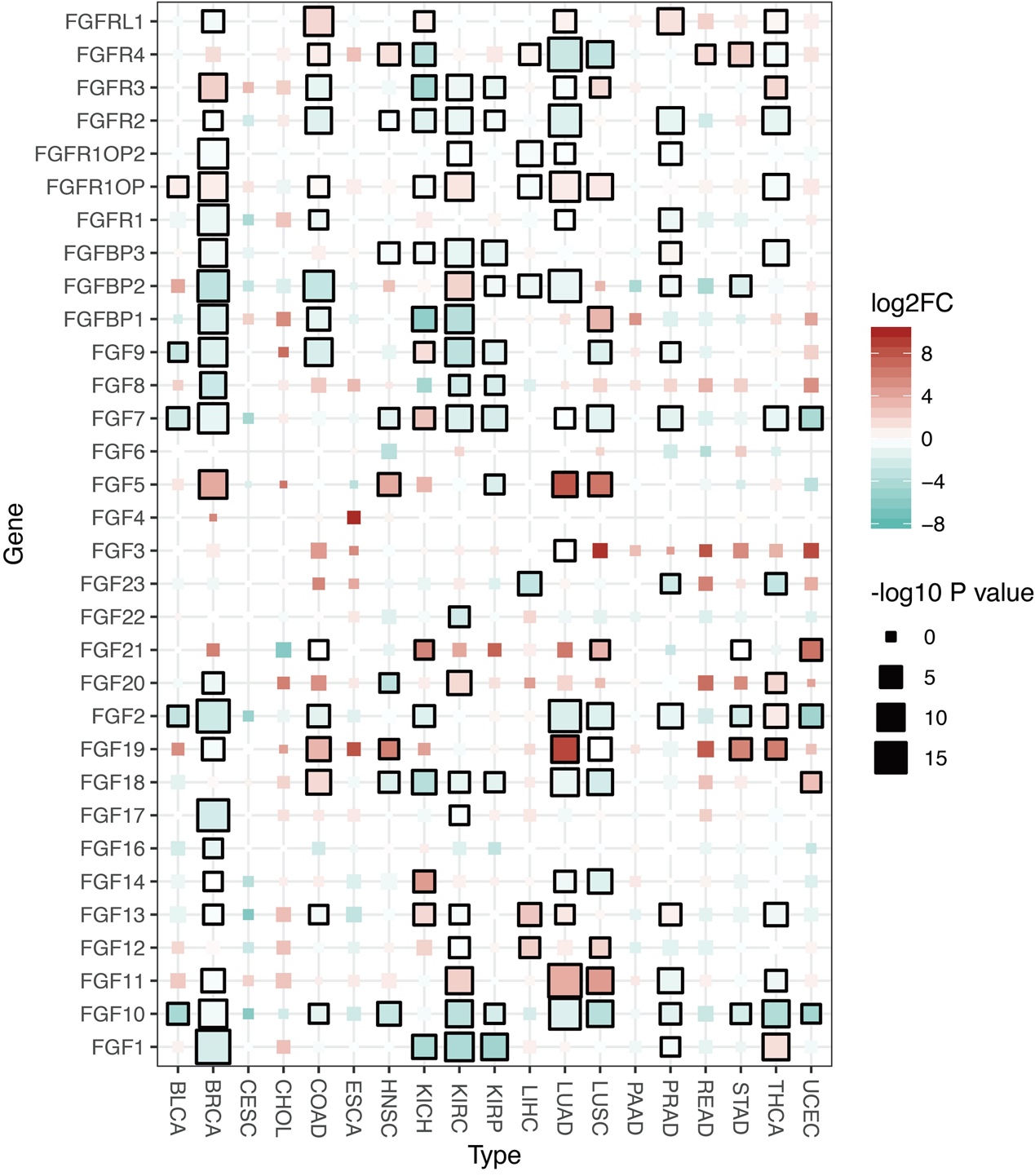


Supplementary Figure S1. ratea


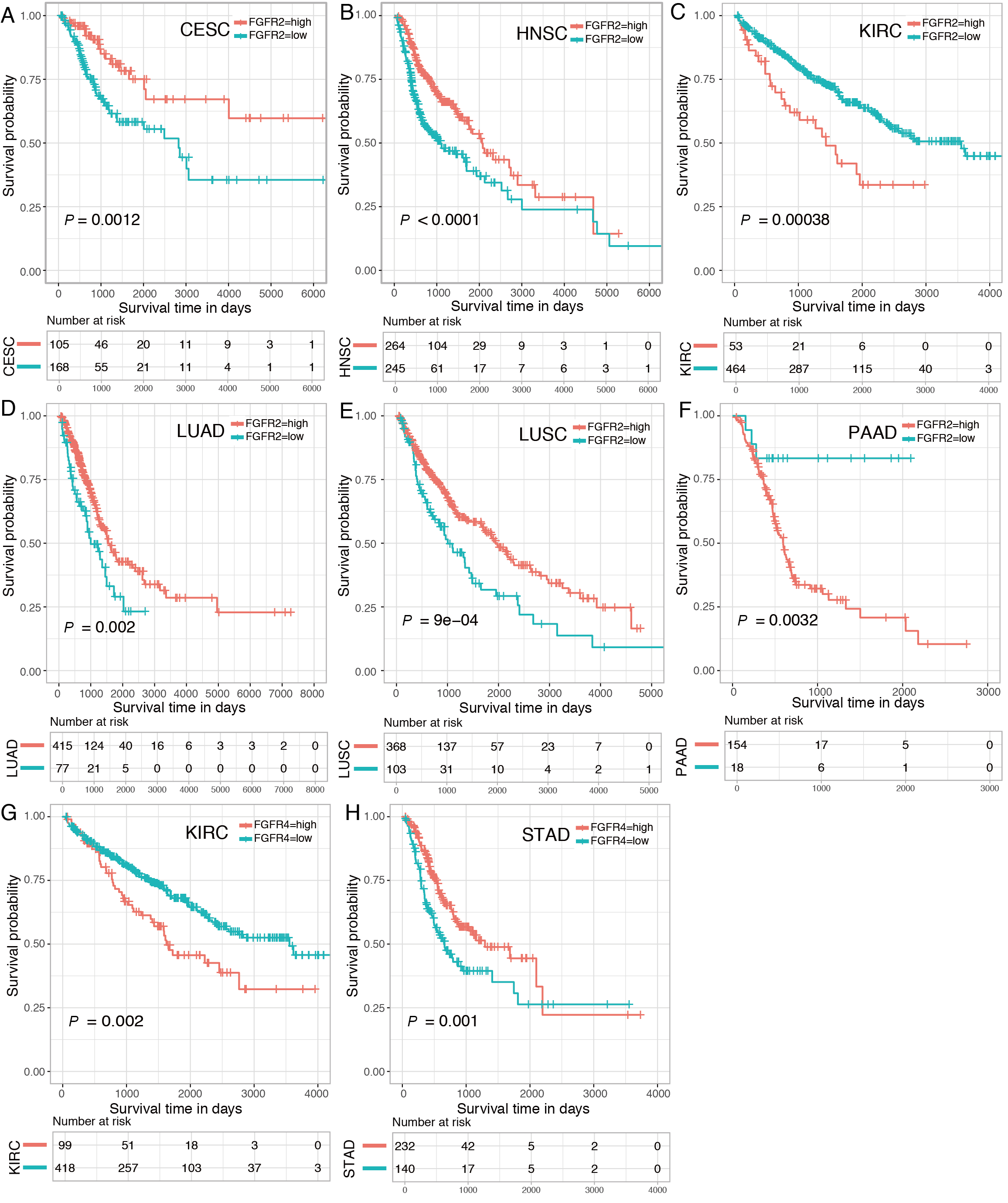


Supplementary Figure S2. Kaplan-Meier plots of FGFR 2 or 4 in specific tumor types.


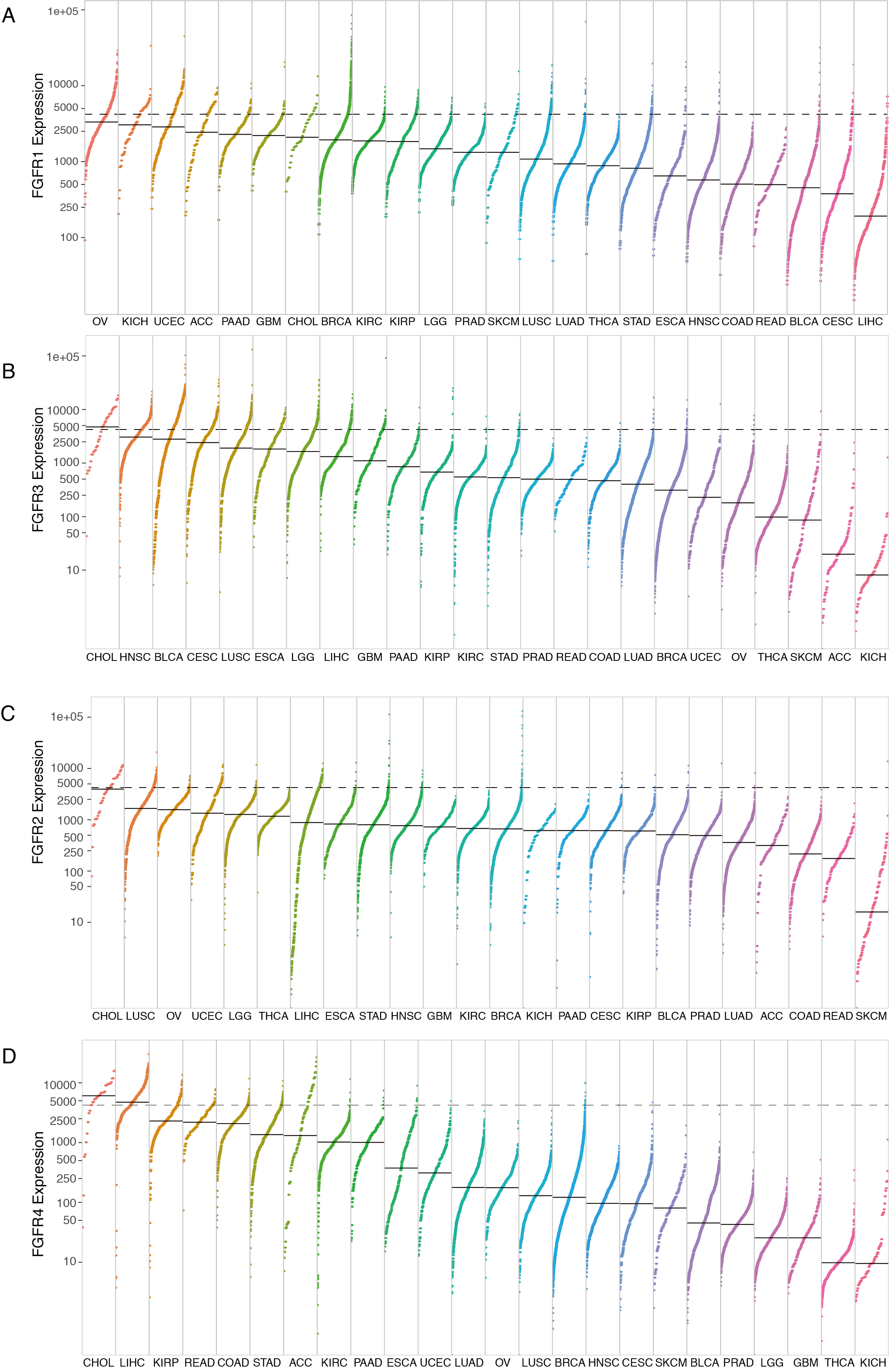


Supplementary Figure S3. The specific expression of FGFR1-4 for each patients in pan-cancer.


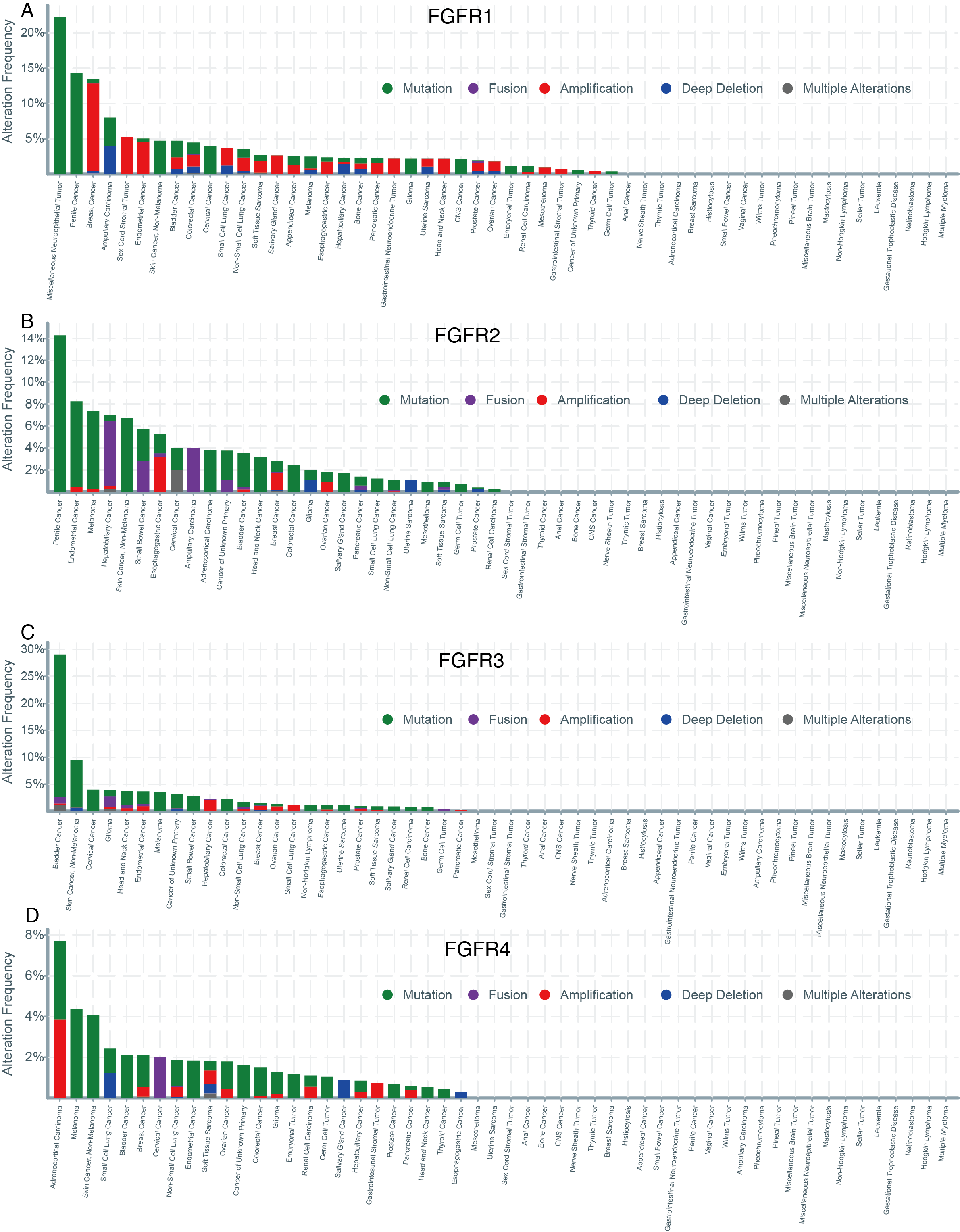


Supplementary Figure S4. The mutation frequencies of FGFR1-4 in pan-cancer from cBioPortal.
